# Surgical placement of left ventricular lead for cardiac resynchronisation therapy after failure of percutaneous attempt

**DOI:** 10.5830/CVJA-2016-046

**Published:** 2017

**Authors:** Mehmet Ezelsoy, Suleyman Yazici, Ertan Sagbas, Muhammed Bayram, Nuran Yazicioglu

**Affiliations:** Department of Cardiovascular Surgery, Bilim University, Istanbul, Turkey; Department of Cardiovascular Surgery, Bilim University, Istanbul, Turkey; Department of Cardiovascular Surgery, Bilim University, Istanbul, Turkey; Department of Cardiovascular Surgery, Mehmet Akif Ersoy Hospital, Istanbul, Turkey; Cardiology, Florence Nightingale Hospital, Istanbul, Turkey

**Keywords:** cardiac resynchronisation therapy, surgically placed epicardial left ventricular lead, heart failure

## Abstract

**Objective:**

Cardiac resynchronisation therapy has been shown to be an effective treatment to improve functional status and prolong survival of patients in advanced chronic heart failure. This study assessed the surgical outcomes of left anterior mini-thoracotomy for the implantation of left ventricular epicardial pacing leads in cardiac resynchronisation therapy.

**Methods:**

Our study consisted of 30 consecutive patients who underwent cardiac resynchronisation therapy with a left thoracotomy between November 2010 and April 2012 in our clinic. Postoperative follow up included the assessment of New York Heart Association (NYHA) functional class, electrocardiography and echocardiography.

**Results:**

There were 22 male and eight female patients with a mean age of 68 ± 5.04 years. All patients were in NYHA class III or IV. Pre-procedure mean left ventricular ejection fraction was 28.1 ± 4.5% and post-procedural ejection fraction improved to 31.7 ± 5.1%. The pre-operative QRS duration changed from 171.7 ± 10.8 to 156.2 ± 4.4 ms after the operation. Also there was a significant reduction in left ventricular end-diastolic dimension from 6.98 ± 0.8 to 6.72 ± 0.8 mm (p < 0 .05), but no change in left ventricular end-systolic dimension and severity of mitral regurgitation. All patients had successful surgical left ventricular lead placement. There was no procedure-related mortality. The mean follow-up time was 40.4 months.

**Conclusion:**

Surgical epicardial left ventricular lead placement procedure is a safe and effective technique in patients with a failed percutaneous attempt.

## Objective

Cardiac resynchronisation therapy (CRT) improves the symptoms of congestive heart failure (CHF), increases exercise tolerance and decreases rates of hospital readmission. Furthermore, CRT improves ejection fraction and survival rate.^1^ Most of these data have been derived in large trials using a transvenous approach, placing the left ventricular lead via the coronary sinus (CS).

While this approach is least invasive, it can be challenging due to restriction of the coronary sinus anatomy, epicardial scar, and unintended stimulation of the left phrenic nerve.^2^ Due to these restrictions, success rates of the percutaneous approach are 75 to 93%.^3^ We aimed to evaluate the surgical outcomes of left anterior mini-thoracotomy for the implantation of left ventricular epicardial pacing leads for CRT after failure of a percutaneous attempt.

## Methods

The ethics committee of Istanbul Bilim University approved this study, which consisted of 30 consecutive patients who underwent surgical placement between November 2010 and April 2012 of a left ventricular (LV) lead with a left thoracotomy after failure of the percutaneous attempt. This study included patients with New York Heart Association (NYHA) functional class III or IV heart failure, ischaemic (25%) or non-ischaemic cardiomyopathy (75%) with a left ventricular ejection fraction (LVEF) ≤ 35% and QRS duration > 120 ms.

Pre- and postoperative follow up involved assessment of NYHA functional class, electrocardiography (ECG), determination of QRS duration, and echocardiographic data. LVEF, left ventricular end-diastolic dimension (LVEDD) and severity of mitral regurgitation (MR) data were collected to analyse the effect of CRT via epicardial LV lead placement on reverse ventricular remodelling. The procedures followed were in accordance with institutional guidelines.

A mini-thoracotomy was performed under deep sedation with no need for selective intubation. The patients were placed in a 45° rotation to the right side. A 3- to 4-cm long left minithoracotomy was performed through the fourth intercostal space between the anterior and mid-axillary line. The pericardium was opened longitudinally anterior to the phrenic nerve and suspended with traction sutures to better expose the lateral wall. Epicardial leads were implanted posterior to an obtuse marginal branch, avoiding areas of scarred myocardium.

Once a site with satisfactory pacing threshold was identified (impedance > 200 Ω and < 2 000 Ω, sensing > 5 mV and pacing threshold measured at 0.5 ms < 2.0 V), the lead was sewn with 5/0 polypropylene sutures. The connector of the lead was tunnelled submuscularly to the device pocket and the pacemaker. Patients were generally extubated in the operating room and observed in the cardiac surgery recovery unit.

## Statistical analysis

SPSS 21.0 software (SPSS Inc, Chicago, IL, USA) was used for the statistics. For data processing, besides descriptive statistical methods such as frequency, percentage, mean values and standard deviation, the Kolmogorov–Smirnov test was used to evaluate the data distribution. For comparison of the parameters in specific groups, the Wilcoxon Z-test and kappa analysis were used. Survival analysis was obtained with the Kaplan–Meier method. The results were evaluated for significance (p < 0 .05).

## Results

Between November 2010 and April 2012, 30 patients (75% male) with a mean age of 68 ± 5.04 years underwent epicardial LV lead placement following a failed attempt at percutaneous CRT. Table 1 summarises the baseline demographics for the patients included in this study.

**Table 1:** Baseline clinical demographics of patients

*Variables*	*Number (%)*
Gender	68 ± 5.04
Male	2 (75)
Female	8 (25)
Aetiology	
Non-ischaemic cardiomyopathy	8 (25)
Ischaemic cardiomyopathy	22 (75)
Co-morbidities	
Diabetes mellitus	13 (43)
Hypertension	18 (60)
Previous myocardial infarction	17 (56)
Chronic obstructive pulmonary disease	8 (26)
Chronic renal failure	9 (30)
Previous cardiac surgery	7 (23)
Previous pacemaker/ICD	10 (33)

**Fig. 1.**
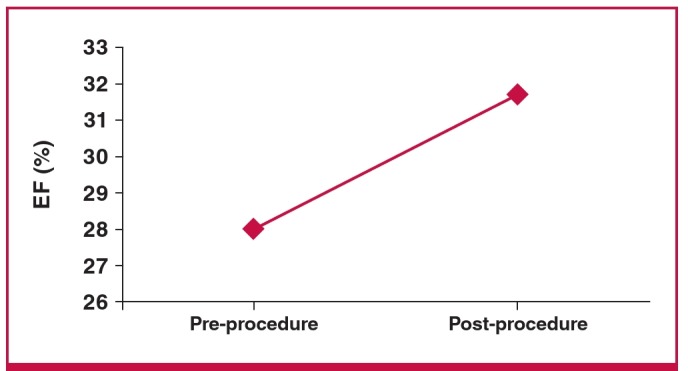
Pre- and post-procedural mean left ventricular ejection fraction.

All patients were in NYHA functional class III or IV. Pre-procedure mean LVEF was 28.1 ± 4.5% and ejection fraction improved to 31.7 ± 5.1% post procedure (Fig. 1).

**Fig. 2.**
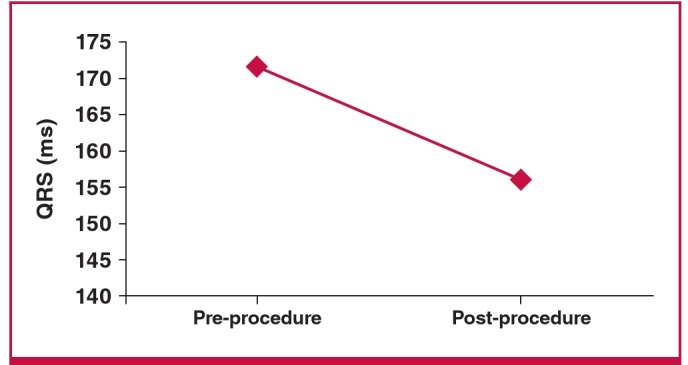
Pre- and post-procedural mean QRS duration.

The pre-surgery QRS duration reduced from 171.7 ± 10.8 to 156.2 ± 4.4 ms post surgery (Fig. 2). In addition there was a significant reduction in LVEDD, from 6.98 ± 0.8 to 6.72 ± 0.8 mm (p < 0 .05), but no change in left ventricular end-systolic dimension (LVESD) and in severity of MR (p > 0 .05) (Table 2).

**Table 2:** Clinical and echocardiographic outcomes following surgical lead placement

*Parameters*	*Pre-procedural outcome*	*Post-procedural outcome*	*p-value*
LVEDD (mm)	6.98 ± 0.8	6.72 ± 0.8	0.030
LVESD (mm)	5.97 ± 0.8	5.90 ± 0.8	0.128
EF (%)	28.1 ± 4.5	31.7 ± 5.1	0.000
Moderate or severe MR, n (%)	6 (42)	7 (43)	0.080
QRS (ms)	171.7 ± 10.8	156.2 ± 4.4	0.000

LVEDD, left ventricular end-diastolic dimension; LVESD, left ventricular end-systolic dimension; EF, ejection fraction; MR, mitral regurgitation.

Patients spent an average of 1.3 ± 0.4 days in the intensive care unit post operation. Mean length of hospital stay was 4.9 ± 2.2 days. Mean duration (skin-to-skin) of procedure was 52.6 ± 12.5 minutes for left ventricular lead implantation through the mini-thoracotomy.

All patients had successful surgical LV lead placement. There was no procedure-related mortality. Intravenous therapy was commonly administered, with diuretics used in 92% of patients and inotropes in 10% of patients.

**Fig. 3.**
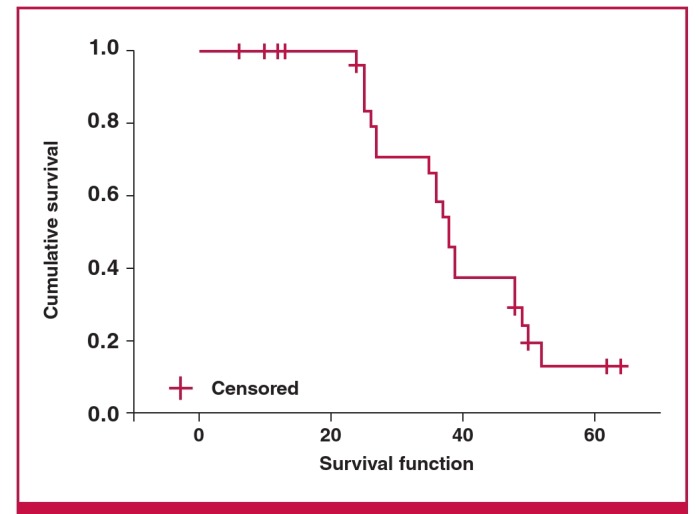
Long-term survival following surgical left ventricular lead placement by Kaplan–Meyer analysis (n = 30).

In total, one patient underwent heart transplantation within five months of surgical lead placement. Ten patients (30%) died during the observation period. The mean follow-up time was 40.4 months (Fig. 3).

## Discussion

CRT has been well-documented to improve left ventricular ejection fraction, heart failure symptoms and survival.^2^ A percutaneous transvenous approach for CRT depends on several factors, such as coronary sinus anatomy, and it can be time consuming.^4^ If there are small coronary veins, it may not be feasible, whereas in the case of large coronary veins, it is often associated with changes in pacing threshold. Furthermore, lifethreatening complications such as coronary sinus perforation may occur.^5^ Sub-optimal LV lead positioning may lead to unfavourable clinical outcomes following CRT.

The advantage of surgical epicardial LV lead positioning is that direct visualisation helps to select the most suitable surface and avoid epicardial fat or fibrosed areas, which can cause changes in pacing thresholds. Mair et al.^6^ recommend that CS lead implantation should be stopped if the procedure exceeds two hours. In our cases, it took 52.6 ± 12.5 minutes from skin incision to completion of LV lead implantation.

In terms of lead function, the pacing threshold in our patients was lower or comparable to that of CS leads.^6^ We acknowledge that the pacing threshold of epicardial leads may increase over time due to myocardial fibrosis, and this may lead to early battery replacement. However, there is little information on longterm follow up of LV lead threshold in CRT. Further studies with a longer follow up are essential.

Transvenous insertion of LV leads is currently the route of choice for CRT. Unfortunately, its success rate is about 75 to 93%, as it is totally dependent on the inconsistent coronary venous anatomy.^6^ Although some centres do describe excellent success rates with percutaneous leads, this does not appear to reflect the average experience. Early and late implantation failures are reported to occur in about 15 and 10% of patients, respectively, with inability to cannulate the coronary sinus being the most frequent reason for failure of lead implantation.^6^

In the original reports of CRT, epicardial LV leads were placed surgically via a left thoracotomy. These procedures were associated with high apparent success rates.^7^ One small trial demonstrated that surgical placement of epicardial LV leads improved symptoms as well as CS lead placement at six months. It is not known if epicardial LV lead placement after a failed transvenous percutaneous approach improves survival or symptoms in the long term.^8^

Puglisi et al.^9^ reviewed their experience with epicardial LV lead placement via a limited left thoracotomy in 33 patients with failed transvenous lead implantation or who had experienced early lead dislodgement. Similar to our results, they found a larger proportion of idiopathic heart failure in patients undergoing thoracotomy compared with patients who had successful percutaneous CRT, and no significant reduction in MR. They reported no surgical complications, optimal lateral lead position in all patients, and five late deaths (15%).

Similarly, we had no surgical complications. In our study we observed that 10 patients in NYHA functional class IV died at the time of percutaneous implantation.

Mair et al.^10^ described a cohort of 80 patients who had successful LV lead implantation by thoracotomy, video-assisted thoracoscopy, or robotically enhanced manipulation. Although no serious adverse events were reported, technical failures occurred in a minority of cases. Others have reported successful CRT with video-assisted thoracoscopic surgery and robotassisted approaches.^11^,^12^

Putnik et al.^13^ reviewed the reduction in QRS complex width (to 26.25 ms) and the increase in LVEF (12.2%). Similarly, in our study we also described reduction in QRS complex and LVEF improvement. We reviewed our surgical experience and found that elective epicardial LV lead placement was associated with improved functional status similar to that demonstrated with transvenous LV lead placement.^2^

In our study, a greater percentage of patients referred for epicardial LV lead placement after a failed coronary sinus approach had non-ischaemic heart failure, which suggests that heart failure aetiology may be predictive of failure of transvenous CRT. It is possible that a greater degree of cardiac chamber and/ or coronary sinus enlargement in patients with non-ischaemic cardiomyopathy may limit access to appropriate pacing sites via the coronary sinus, although this remains to be proven. By contrast, the presence of scarred myocardium may be more likely to lead to unacceptable pacing and sensing thresholds in patients with ischaemic cardiomyopathy.

Our results of epicardial LV lead placement demonstrate a clear advantage of avoiding lead-related complications and the necessity of re-operations. Surgical LV lead placement offers the advantage of direct access to the lateral left ventricular wall. Direct visualisation provides an almost unrestricted opportunity to implant the leads at the optimal target site, so that the pre-determined lead position was achieved in all patients.

Our analysis is limited by small sample size, lack of data regarding ventricular capture post implantation and the retrospective design.

## Conclusion

The mini-thoracotomy approach for left ventricular lead implantation is feasible and may avoid some of the limiting factors of transvenous procedures. Furthermore, our observed early functional and haemodynamic improvements show a similarity with that in the literature. This method allows optimal lead implantation under direct vision and therefore reduces the incidence of non-responders, resulting from sub-optimal lead placement. We believe that with improvement in epicardial leads, it may even have potential benefits as primary intervention in a specific subset of patients. With further development of minimally invasive surgical techniques and refinement in choice of pacing leads and lead positions, epicardial left ventricular lead placement may become a reasonable alternative for select patients with heart failure.
